# The additive effect of biochar amendment and simulated nitrogen deposition stimulates the plant height, photosynthesis and accumulation of NPK in pecan (*Carya illinoinensis*) seedlings

**DOI:** 10.1093/aobpla/plaa035

**Published:** 2020-07-26

**Authors:** Zhiying Hou, Yiquan Tang, Caiyun Li, Kean-Jin Lim, Zhengjia Wang

**Affiliations:** State Key Laboratory of Subtropical Silviculture, Zhejiang A&F University, Hangzhou, Zhejiang, China

**Keywords:** Carbon cycling, nitrogen retention, nutrient absorption, soil bulk density, soil enzyme activity

## Abstract

This work investigated the effective doses of biochar (BC) amendment with simulated nitrogen deposition on the stimulation of pecan (*Carya illinoinensis*) growth. A total of nine conditions combining three levels of BC—BC0, 0 t ha^−1^ year^−1^; BC20, 20 t ha^−1^ year^−1^; and BC40, 40 t ha^−1^ year^−1^—and three levels of simulated nitrogen deposition—N0, 0 kg N ha^−1^ year^−1^; N50, 50 kg N ha^−1^ year^−1^; and N150, 150 kg N ha^−1^ year^−1^—were applied throughout 1 year on the pecan-grafted seedlings of cultivar ‘Pawnee’. The growth, photosynthesis, chlorophyll and nutrient content in the seedlings were measured. The soil bulk density, pH, nitrogen content and enzymatic activities were also measured. Biochar amendment reduced soil bulk density and elevated soil pH. Meanwhile, aided by BC amendment, the inorganic nitrogen content and enzyme activities increased with increasing doses of nitrogen. In the absence of BC amendment, the seedlings’ height, photosynthesis and chlorophyll pigments were only stimulated by a low level of simulated nitrogen deposition (N50), whereas a high level of simulated nitrogen deposition (N150) impeded the growth. The seedlings improved the most under the combined treatment of BC20N150, wherein the seedling heights, photosynthesis and total chlorophyll improved by 22 %, 70 % and 40 %, respectively, compared to those treated solely with BC20. Further increase of nitrogen retention in the soil by the BC40 did not further improve the growth of the seedlings, suggesting the possible mechanisms involve nutrient uptake and usage dynamic in the seedlings. The BC amendment alleviated the antagonist effect from simulated nitrogen deposition that suppressed the absorption of phosphorus, potassium and iron. The effect of applying both BC amendment and simulated nitrogen deposition to the growth of seedlings was additive at fertilizing tree species.

## Teaser

This work investigated the effect of combining biochar (BC) amendment and simulated nitrogen deposition on the growth of pecan seedlings at varying doses. Under the combined treatment of a low level of BC amendment and a high level of stimulated nitrogen deposition, the seedlings’ height, photosynthetic ability and total chlorophyll content improved the most compared with BC treatment alone. The BC amendment alleviated the antagonist effect from simulated nitrogen deposition that suppressed the absorption of phosphorus, potassium and iron. The effect of applying both BC amendment and simulated nitrogen deposition to the growth of seedlings was additive at fertilizing tree species.

## Introduction

Increasing the nutrient availability and efficiency of nutrient use in plants have been researched to reduce the adverse environmental impacts caused by overuse of commercial fertilizer. Biochar (BC), a soil modifier derived from high-temperature treatment of crop stalks, dead branches, pericarp shells, etc. (Antal and Grønli 2003) has been widely used to improve soil health, thereby promoting plant growth (Jeffery *et al.* 2011). Due to its low density, high porosity and carbon-rich content, numerous studies have found that application of BC can aerate compact soil, decreasing soil bulk density and revamping microbial community composition (Xiao *et al.* 2017; Palviainen *et al.* 2018; Zhao *et al.* 2018). In addition to its rich carbon content (>60 % carbon), BC is rich in nutrients such as calcium, potassium, magnesium, nitrogen and phosphorus (Wang *et al.* 2014); thus, it can be utilized to improve soil nutrients, promote root growth and nutrient absorption and increase plant biomass and yield (Van Zwieten *et al.* 2010; Taghizadeh-Toosi *et al.* 2012; Bornø *et al.* 2018). In addition, a study by Agegnehu *et al.* (2016) revealed that adding BC was effective at increasing the chlorophyll content of wheat leaves and slowing down leaf senescence, indicating that BC could be utilized as a potential soil modifier to improve plant fitness.

Nitrogen is lost from soil in the form of NH_4_ and NO_3_ through leaching or volatilization (Steiner *et al.* 2008). Settling of atmospheric NH_4_ and NO_3_ via nitrogen deposition into terrestrial and aquatic ecosystems has caused a series of ecological and environmental problems. It was estimated that the global atmospheric nitrogen deposition flux will continue to increase to 194.5 Tg N year^−1^ by 2050 (Galloway *et al.* 2004), and overapplication of commercial fertilizer is one of the main causes of the increase of nitrogen emissions. Biochar influences soil nitrogen transformations and has been shown to mitigate N_2_O emissions by influencing nitrification rates in the field (Clough and Condron 2010). The effect of BC on the availability of soil nitrogen arises from its cation exchange capacity (CEC), which enhances inorganic nitrogen storage in the soil (Cao *et al.* 2018a). Research in an experimental field located in the central Amazon found that, following secondary forest clearing, soil amendment of BC was effective at enhancing nitrogen retention through absorption and cation exchange mechanisms as well as changes to microbial composition that affected the enzyme dynamic in the soil (Clough and Condron 2010). Adding BC to the soil also increases mineralization and/or ammonia oxidation in the soil (Dempster *et al.* 2011; Maestrini *et al.* 2014). Soil acidification caused by the settling of atmospheric nitrogen via the introduction of anions and H^+^ will lead to increased availability of toxic cations such as aluminium, iron, etc. (Bowman *et al.* 2008; Phoenix *et al.* 2012; Zhang *et al.* 2017). Nonetheless, such soil toxicity can be overcome by BC amendment due to its alkalinity and high CEC, which counteracts reduced uptake of trace elements in plants (Rizwan *et al.* 2016).

To date, the studies of the benefits of BC amendment with simulated nitrogen deposition have mainly focused on agricultural crops, such as corn (Bornø *et al.* 2018) and rice (Makino *et al.* 2000), while study of tree species’ growth is still lacking (Currie and Nadelhoffer 1999; Glaser *et al.* 2002). Zhang *et al.* (2017) revealed that, aided by BC amendment, the nut quality and soil fertility of *Torreya grandis*, a conifer belonging to the Taxaceae family, could be improved with simulated nitrogen deposition. Studies on the interaction of dicotyledon species with soil amendment by BC modification and simulated nitrogen deposition remain rare. Pecan (*Carya illinoinensis*), a dicot belonging to the Juglandaceae family, is highly popular for its nutritional value and taste (Han *et al.* 2018). It can also be used for ecological protection and environmental greening; thus, it is both an economically and ecologically important tree species (Venkatachalam *et al.* 2007). Nitrogen fertilization is important to maintain the growth of pecan plants and their nut production (Wells 2013). The application of nitrogen fertilizer for pecans is commonly carried out in the spring, while nitrogen application in the fall has only been effective for certain cultivars (Smith *et al.* 1995), indicating the response of pecans to nitrogen fertilization can vary according to genotype or seasonal changes. The response of pecans to the application of BC with simulated nitrogen deposition remains unknown. In this work, we studied the effect of BC amendment with varying levels of nitrogen deposition on the growth of pecan seedlings. The physicochemical properties, enzyme dynamic in the soil, photosynthetic efficiency and accumulation of nutrients in the seedlings were studied. The incorporation of BC amendment and nitrogen deposition into fertilizer management would be a step closer to reducing the use of fertilizer in the field, which has been problematic for the environment.

## Materials and Methods

### Plant material, study site and sampling timing

The experiment was conducted using 1-year-old grafted pecan seedlings of the cultivar ‘Pawnee’ at the greenhouse located at east longitude 119°43′38″ and north latitude 30°15′16″ from April 2017 to May 2018. Inside the greenhouse, light transmittance was 80–85 %, temperature was 30/20 °C (day/night) and relative humidity was 70–80 %. The seedlings were transplanted in the pots of volume 16 L that filled with soil, perlite and organic fertilizer mixture (3:1:1, v/v/v), and they were watered daily. After growing in the standardized condition for 2 months, a total of 81 seedlings of similar height and growth conditions were selected as the test material. The growth index and photosynthesis parameters of the seedlings were measured and leaf samples were collected for biochemical studies in May, July, September and November 2017 and May 2018.

### Biochar treatment and nitrogen deposition

The BC was derived from pyrolysis of wheat straw at 450 °C under anoxic condition (Sanli New Energy Company, Henan, China). The crude BC material was ground and screened through a 2-mm sieve to obtain particles in high consistency. The final physicochemical properties of the BC material were: pH: 9.80; bulk density: 0.5 g cm^−3^; specific surface area: 9.7 m^2^ g^−1^; CEC: 189.3 cmol kg^−1^; organic carbon: 425 g kg^−1^; total N: 5.2 g kg^−1^; total P: 3.4 g kg^−1^; ash: 18.6 % (Zhang *et al.* 2017). The nitrogen deposition was simulated by dissolving ammonium nitrate (NH_4_NO_3_) in water and spraying it on leaves and soil. The amount of nitrogen deposition was proportioned according to the annual precipitation of 1628.6 mm in the Lin’an district. Three levels of BC—BC0, 0 t ha^−1^ year^−1^; BC20, 20 t ha^−1^ year^−1^; and BC40, 40 t ha^−1^ year^−1^—and three levels of simulated nitrogen (N) deposition—N0, 0 kg N ha^−1^ year^−1^; N50, 50 kg N ha^−1^ year^−1^; and N150, 150 kg N ha^−1^ year^−1^—were applied. The pH of N0, N50 and N150 was 6.92, 5.97 and 5.65, respectively. The seedlings were divided into nine groups containing nine plants, and each group was treated with a different condition—BC0N0 (control), BC0N50, BC0N150, BC20N0, BC20N50, BC20N150, BC40N0, BC40N50 and BC40N150—and pooled into three biological replicates.

### Growth index of pecan-grafted seedlings

Plant height was determined by measuring the length of the main stem from base to tip using a meter ruler with a resolution of 0.1 cm. The stem diameter was determined by measuring 5 cm above the graft interface using a vernier calliper with a resolution of 0.01 mm. The diameter was measured at three points and an average value was obtained.

### Determination of photosynthetic gas exchange parameters

The photosynthetic parameters included the net photosynthetic rate (*A*, μmol m^−2^ s^−1^), stomatal conductance (*g*_s_, mol m^−2^ s^−1^), intercellular CO_2_ concentration (*C*_i_, μmol m^−2^ s^−1^) and transpiration rate (*E*, mmol m^−2^ s^−1^) were determined using a Licor Li-6400XT portable photosynthesis system at 800 μmol m^−2^ s^−1^ light intensity, 60–70 % relative humidity and 500 μmol s^−1^ flow rate. The CO_2_ concentration in the reference chamber was controlled at 390 ± 10 μmol L^−1^, and the chamber temperature was 25 ± 1 °C. The measurement was performed on sunny days from 0900 to 1100 h. Matured fresh leaves under good light conditions were selected for measurement. Leaves were kept in the chamber for 3–5 min to stabilize the gas exchange before recording the readings.

### Determination of chlorophyll and carotenoid content

Fully expanded fresh leaves were randomly collected and brought back to the laboratory. Leaves were washed, dried and cut into small pieces. An amount of 0.1 g was added into a container containing 10 mL of 95 % ethanol and soaked for 45 h in the absence of light (or until the leaves turned white). The absorbance values of the ethanol extract were measured at wavelengths of 470, 646 and 663 nm using a UV-VIS spectrophotometer (Shimadzu Co., Ltd, China). The chlorophyll a (Chla), chlorophyll b (Chlb), total chlorophyll (Chl) and carotenoid (Car) content per gram of leaves were calculated according to the Arnon formula (Arnon 1949).

### Determination of nutrient elements in leaves of pecan seedlings

The leaf samples were baked at 105 °C for 30 min for green killing. They were then dried at 70 °C until constant weight was obtained, then ground into a fine powder. An amount of 0.2 g leaf powder was added into a test tube and wet with MiliQ water, then 5 mL of concentrated sulfuric acid (H_2_SO_4_) was added. The mixture was left to stand on a bench overnight. Sample digestion was performed the next day using an EHD36-DigiBlock Digester (Lab Tech, China). The N content was determined by the Kjeldahl method according to the procedures described by Ma *et al.* (2018). The phosphorus (P) content was determined by the molybdenum-ruthenium colorimetry method described by Cao *et al.* (2018b). The content of the metal elements calcium (Ca), copper (Cu), iron (Fe), potassium (K), magnesium (Mg), manganese (Mn) and zinc (Zn) was determined according to the procedures described by Stewart and Růžička (1976), using a PE-2100 atomic absorption spectrophotometer (Beijing Jingke Ruida Technology Co., Ltd, China).

### Determination of soil bulk density and pH

The soil samples were extracted with a ring knife and dried in an oven at 110 °C to obtain the dry weight. Soil bulk density was calculated according to the following formula: (*W*1 − *W*2)/*V* (*W*1: mass of ring knife after filled with soil [g]; *W*2: mass of empty ring knife [g]; *V*: volume of ring knife [cm^3^]). For the determination of soil pH, a volume of 25 mL distilled water was added into a beaker containing 10 g dried soil (water to soil ratio 2.5:1). The mixture was stirred with a magnetic stirrer for 1 min, let stand for 30 min and the pH was measured with a pH meter.

### Determination of soil’s inorganic nitrogen content

The soil’s inorganic nitrogen was present in the form of ammonium nitrogen (NH_4_^+^-N) and nitrate nitrogen (NO_3_^−^-N). To determine the NH_4_^+^-N content, a 5 g soil sample was first extracted with 25 mL 2 M potassium chloride (KCl), and 10 mL of the soil extract was added into a 50-mL volumetric flask. Subsequently, 5 mL 0.1 M phenol solution was added, which was then followed by 5 mL sodium hypochlorite (NaClO) solution. The mixture was mixed vigorously and stood at room temperature for an hour. A volume of 1 mL masking reagent containing 1.4 M potassium sodium tartrate (C_4_H_4_KNaO_6_) and 0.3 M ethylenediaminetetraacetic acid (EDTA) was added to remove any visible precipitate, and then topped up to 50 mL using MiliQ water. The absorbance value at wavelength of 625 nm was obtained using a UV-VIS spectrophotometer (Shimadzu Co., Ltd, China; Krom 1980). The NH_4_^+^-N content in the soil extract was determined against the standard curve established using ammonium sulfate ((NH_4_)_2_SO_4_).

The measurement of NO_3_^−^-N content was based on determining its characteristic UV absorption at 220 nm. Due to organic matter also exhibiting UV absorption at 220 and 275 nm, the absorbance at 275 nm (no UV absorption by NO_3_^−^) was obtained to subtract the contribution from interfering organic matter. An amount of 10 g soil sample was extracted with 100 mL deionized water containing 0.2 g CaSO_4_. The mixture was rotated in a shaker for 15 min and stood for 30 min, and the supernatant was filtered. Then 1 mL 1 M hydrochloric acid (HCl) was added to a test tube containing 50 mL filtrate. The absorbance value at wavelength of 210 nm was obtained using a UV-VIS spectrometer. The NO_3_^−^-N content in the filtrate was determined against the standard curve established using NO_3_^−^-N standard.

### Determination of soil enzyme activity

The enzyme activity of invertase, urease, cellulase and acid phosphatase in the soil was measured according to the methods described by Guan (1986). Briefly, the measurement of enzymatic activity of invertase, urease, cellulase and acid phosphatase was conducted using a substrate: sucrose, urea solution, carboxymethylcellulose and benzene disodium phosphate, respectively. One unit of enzyme activity was expressed by the amount of substrate (or product) consumed (or produced) by soil enzymes per unit mass of dry soil per unit time.

### Statistical analysis of data

Analysis of variance was performed on the growth index, photosynthesis parameters, chlorophyll content and nutrient elements of the seedlings using SPSS 22v (www.ibm.com), and a Duncan test was used for *post hoc* analysis to compare means. Lillieford tests were employed to check the normality of variance. Plots were made using GraphPad Prism5 (https://www.graphpad.com/).

## Results

### Physicochemical properties of soil and enzyme activities

**Figure 1.**
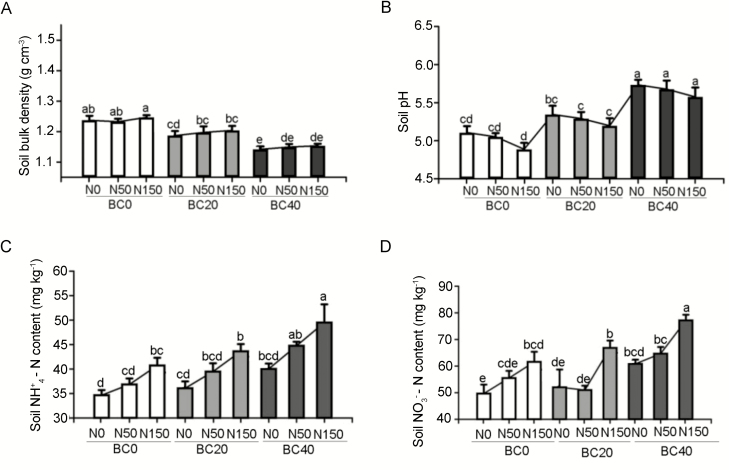
The physicochemical properties of the soil following application of BC amendment and N application: (A) bulk density, (B) pH, (C) NH_4_^+^-N content and (D) NO_3_^−^-N content. BC0: 0 t ha^−1^ year^−1^; BC20: 20 t ha^−1^ year^−1^; BC40: 40 t ha^−1^ year^−1^; N0: 0 kg N ha^−1^ year^−1^; N50: 50 kg N ha^−1^ year^−1^; N150: 150 kg N ha^−1^ year^−1^. Error bars represent standard errors of three biological replicates. Analysis of variance was based on Duncan’s multiple range test. Lowercase letters indicate significance level of *P* < 0.05.

Following application of BC amendment and simulated nitrogen deposition (thereafter named as N application) throughout 1 year, BC amendment improved soil condition, wherein the soil bulk density decreased, and the pH increased with increasing BC dosages (Fig. 1A and B). At the highest level of BC amendment, BC40, the soil bulk density was reduced to 1.15 g cm^−3^, while a weakly acidic soil (pH ~ 6) was attained. In the absence of BC amendment, the soil acidity increased with increasing dosages of N application.

The inorganic N content in the soil was ameliorated by the combined effect of BC amendment and N application (Fig. 1C and D). In the absence of N application, the NH_4_^+^-N and NO_3_^−^-N content in the BC20-modified soil was the same as that in soil not subjected to BC amendment (BC0). However, a higher level of BC amendment, BC40, increased the content of NH_4_^+^-N and NO_3_^−^-N in the soil. The addition of BC was effective at increasing the N content in the soil with N application. The effect was most apparent with the highest level of deposited N. At the highest level of N deposition and BC amendment, BC40N150, the NO_3_^−^-N and NH_4_^+^-N content were 49.52 and 77.19 mg kg^−1^, which were, respectively, 21.55 % and 25.29 % higher than in the non-BC-treated soil (BC0N150).

**Figure 2.**
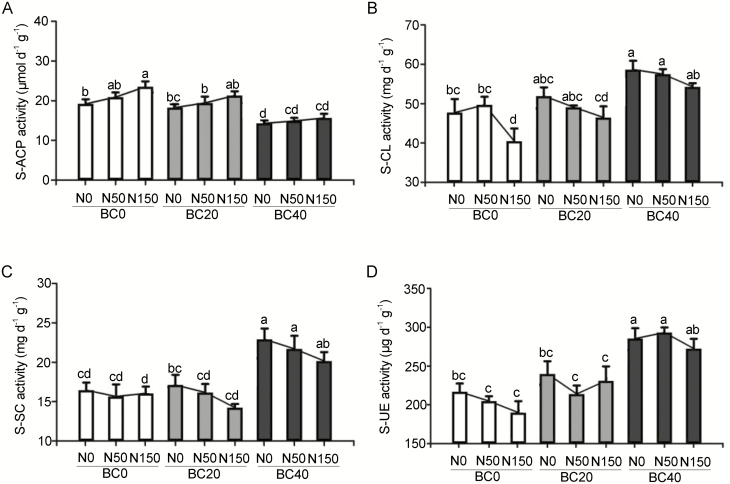
Effect of BC amendment with N application on the soil enzyme activity of (A) acid phosphatase (S-ACP), (B) cellulase (S-CL), (C) sucrose invertase (S-SC) and (D) urease (S-UE). BC0: 0 t ha^−1^ year^−1^; BC20: 20 t ha^−1^ year^−1^; BC40: 40 t ha^−1^ year^−1^; N0: 0 kg N ha^−1^ year^−1^; N50: 50 kg N ha^−1^ year^−1^; N150: 150 kg N ha^−1^ year^−1^. Error bars represent standard errors of three biological replicates. Analysis of variance was based on Duncan’s multiple range test. Lowercase letters indicate significance level of *P* < 0.05.

Soil enzyme activity reflects the nutrient cycling dynamic in the soil. Increasing BC dosages in the soil increased the enzymatic activities of cellulase, invertase and urease, whereas the activity of acid phosphatase decreased (Fig. 2). Increasing dosages of N application slightly affected enzymatic activities: the activity of cellulase, invertase and urease showed a mild downward trend, while the acid phosphatase exhibited a mild upward trend.

### Growth index of pecan-grafted seedlings

**Figure 3.**
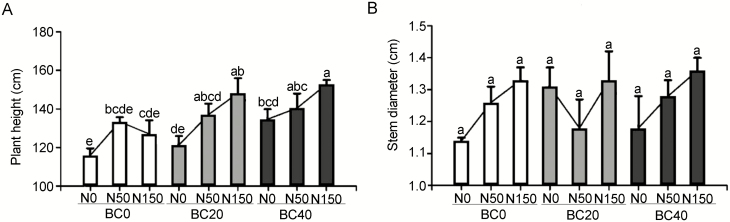
Effect of BC amendment with N application on the (A) height and (B) stem diameter of pecan seedlings. BC0: 0 t ha^−1^ year^−1^; BC20: 20 t ha^−1^ year^−1^; BC40: 40 t ha^−1^ year^−1^; N0: 0 kg N ha^−1^ year^−1^; N50: 50 kg N ha^−1^ year^−1^; N150: 150 kg N ha^−1^ year^−1^. Error bars represent standard errors of three biological replicates. Analysis of variance was based on Duncan’s multiple range test. Lowercase letters indicate significance level of *P* < 0.05.

BC amendment and N application significantly increased seedling height compared to the control seedlings (BC0N0) that were not subjected to BC and N treatment (Fig. 3A; **see****Supporting Information—Table S1**). In the absence of BC (BC0), BC0N50 had the most significant effect on seedling height (*P* < 0.05), and further N deposition with N150 did not improve plant height. Aided by BC amendment, N150 further elevated the plant height of the seedlings. At a treatment of BC20 and BC40, the seedling height treated with N150 advanced the most compared to counterparts treated with BC alone (Fig. 3A; **see****Supporting Information—Table S1**). Notably, BC20N150 and BC40N150 increased seedling height by 22.16 % and 13.53 % (*P* < 0.05) compared to counterparts grown with BC20N0 and BC40N0, respectively (Fig. 3A; **see****Supporting Information—Table S1**), indicating the N application under the influence of BC20 was more effective than the treatment with BC40. The treatment of BC and N application did not affect the growth of stem diameter (Fig. 3B; **see****Supporting Information—Table S1**).

### Photosynthetic parameters in the leaves of pecan seedlings

**Figure 4.**
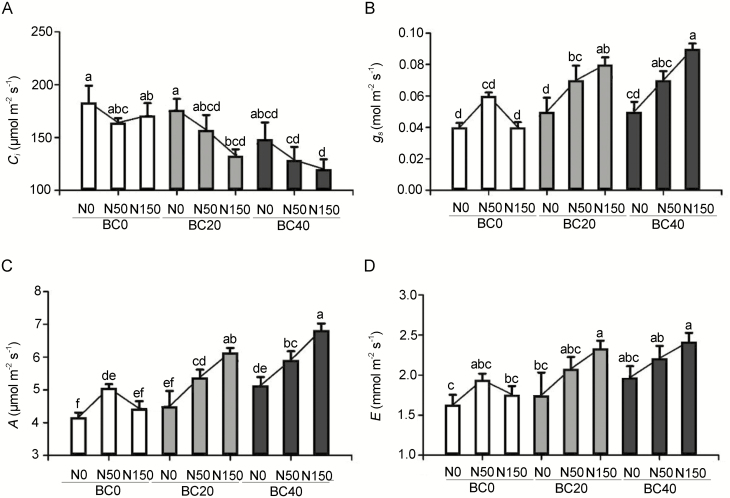
Effect of BC amendment with N application on the (A) intercellular CO_2_ concentration (*C*_i_), (B) stomatal conductance (*g*_s_), (C) net photosynthetic rate (*A*) and (D) transpiration rate (*E*) of the pecan seedlings. BC0: 0 t ha^−1^ year^−1^; BC20: 20 t ha^−1^ year^−1^; BC40: 40 t ha^−1^ year^−1^; N0: 0 kg N ha^−1^ year^−1^; N50: 50 kg N ha^−1^ year^−1^; N150: 150 kg N ha^−1^ year^−1^. Error bars represent standard errors of three biological replicates. Analysis of variance was based on Duncan’s multiple comparison. Lowercase letters indicate significance level of *P* < 0.05.

The photosynthetic ability of the pecan leaves was measured to determine plant fitness when grown with BC amendment and N application. In the absence of BC (BC0), when N application increased, the *g*_s_, *A* and *E* of the leaves increased at first and then decreased, while, conversely, the *C*_i_ decreased first and then increased (*P* < 0.05; Fig. 4; **see****Supporting Information—Table S2**). The BC amendment further enhanced photosynthetic ability under the effect of a high level of N application. Compared to the seedlings treated solely with BC20 and BC40, the *A* of seedlings treated with BC20N150 and BC40N150 increased by 40.44 % and 32.88 % (*P* < 0.05), respectively. Likewise, the *g*_s_ and *E* of the seedlings grown with BC20N150 increased by 70.21 % and 33.71 %, respectively, compared to the seedlings of BC20 alone; the BC40N150 treatment boosted the *g*_s_ and *E* of the leaves by 60.00 % and 22.84 % (*P* < 0.05), respectively, compared to the seedlings treated with BC40 alone. In contrast, the *C*_i_ dropped with the increase of N application under BC20 and BC40 treatment. The *C*_i_ in the leaves of seedlings treated with BC20N150 and BC40N150 was 32.47 % and 23.64 % (*P* < 0.05) of that present in the pecan leaves of BC20N0 and BC40N0, respectively.

### Accumulation of chlorophyll content in pecan leaves

**Figure 5.**
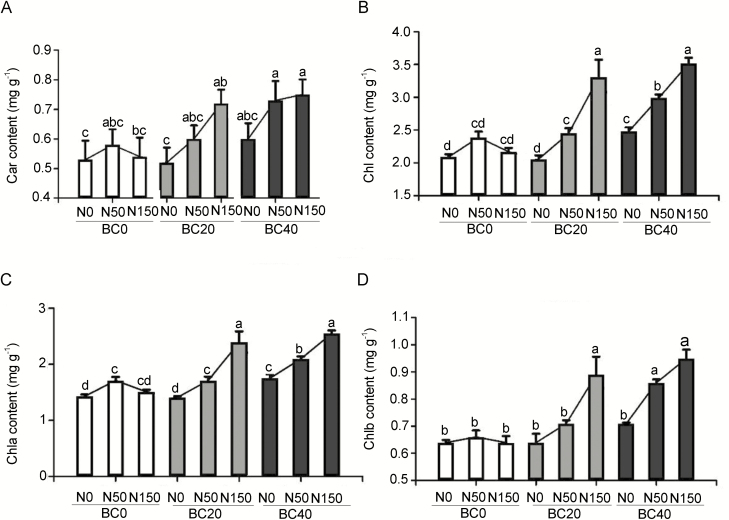
Effect of BC amendment with N application on the content (mg g^−1^ dry weight) of (A) carotenoid (Car), (B) total chlorophyll (Chl), (C) chlorophyll a (Chla) and (D) chlorophyll b (Chlb) in the leaves of pecan seedlings. BC0: 0 t ha^−1^ year^−1^; BC20: 20 t ha^−1^ year^−1^; BC40: 40 t ha^−1^ year^−1^; N0: 0 kg N ha^−1^ year^−1^; N50: 50 kg N ha^−1^ year^−1^; N150: 150 kg N ha^−1^ year^−1^. Error bars represent standard errors of three biological replicates. Analysis of variance was based on Duncan’s multiple range test. Lowercase letters indicate significance level of *P* < 0.05.

Since the combined BC treatment and N application promoted the photosynthetic ability of pecan leaves, it was ultimately important to determine whether the treatment affected the accumulation of chlorophyll in them. At the beginning of BC amendment and N application, no significant effect on chlorophyll content was observed. The chlorophyll content of the leaves increased only after 4 months of treatment **[see****Supporting Information—Table S3****]**. Following 1 year of experiments, the results showed that N application had a significant impact on the accumulation of Chla, which was facilitated by BC20 and BC40 amendment, and the Chla content increased with increasing N deposition (*P* < 0.05; Fig. 5C; **see****Supporting Information—Table S4**). In particular, the enhancement of Chla content by N application with BC20 amendment exceeded those treated with other treatments, when compared to counterparts grown solely with BC amendment. Compared with BC20N0 and BC40N0, the Chla content of BC20N150 and BC40N150 increased by 69.50 % and 45.71 % (*P* < 0.05), respectively (Fig. 5C; **see****Supporting Information—Table S4**). Likewise, N application and BC treatment had similar effects on the Chlb, Chl and Car contents in the leaves (Fig. 5; **see****Supporting Information—Table S4**). Notably, after adding BC20, the effect of N application on the chlorophyll content of pecan leaves was greater than other treatments.

### Accumulation of N and phosphorus content in pecan leaves

**Figure 6.**
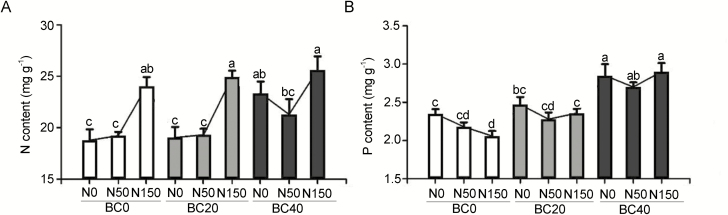
Effect of BC amendment with N application on the content (mg g^−1^ dry weight) of (A) nitrogen (N) and (B) phosphorus (P) in the leaves of pecan seedlings. BC0: 0 t ha^−1^ year^−1^; BC20: 20 t ha^−1^ year^−1^; BC40: 40 t ha^−1^ year^−1^; N0: 0 kg N ha^−1^ year^−1^; N50: 50 kg N ha^−1^ year^−1^; N150: 150 kg N ha^−1^ year^−1^. Error bars represent standard errors of three biological replicates. Analysis of variance was based on Duncan’s multiple range test. Lowercase letters indicate significance level of *P* < 0.05.

The N application in the present work was applied through foliar spraying and soil application. Due to the effects of BC amendment, the content of inorganic N in the soil was enhanced alongside increasing level of N application. We also measured the accumulation of N and phosphorus content in the pecan leaves to learn how the foliar spray affects the nutrient accumulation in pecan leaves. In the absence of N application, a treatment of BC40N0 significantly increased N accumulation in the leaves by 22.52 % and 24.28 % (*P* < 0.05) compared to seedlings grown with BC20N0 and BC0N0, respectively. Upon the onset of N application, we observed that the accumulated N in the leaves of BC20-treated seedlings was no different than those not subjected to BC amendment. For the BC0 and BC20 treatment, the N accumulation was not significantly different in the seedlings treated with a low level of N, N50, compared to those not subjected to N deposition. Nonetheless, the N accumulation was markedly boosted when a high level of N, N150, was applied (Fig. 6A; **see****Supporting Information—Table S5**). The N content in the BC0N150- and BC20N150-treated leaves increased by 25.07 % and 29.03 % (*P* < 0.05) compared with BC0N50 and BC20N50, respectively (Fig. 6A; **see****Supporting Information—Table S5**). Likewise, for the seedlings treated with BC40, the N deposition of 50N was the same as those treated solely with BC40. Meanwhile, a high level of N deposition (N150) increased the N content in the leaf samples by 20 % compared to that treated with BC40N50 (*P* < 0.05; Fig. 6A; **see****Supporting Information—Table S5**).

The elevation of N absorption in the seedlings had somewhat imposed an antagonist effect on the accumulation of P in the leaves. This effect was apparent in the seedlings that were not subjected to BC treatment, for which the P content in the leaves decreased with increasing dosages of N. The P content in the leaves treated with a high level of N (N150) was 14.08 % (*P* < 0.05) lower than those treated with neither BC nor N (Fig. 6B; **see****Supporting Information—Table S5**). The application of BC20 and BC40 seemingly alleviated the retardation of P absorption, especially with the application of a high dosage of N (N150). For the seedlings grown with BC20 and BC40, the phosphorus content in the leaves decreased at first under N0 and N50 treatment, and this negative effect was alleviated with a high dosage of N (N150).

### Accumulation of metal elements in the pecan leaves

The N application and BC amendment also affected the accumulation of metal elements in the pecan leaves. In the absence of N deposition, the metal elements Ca, Cu, K, Mg, Mn and Zn increased with the increase of BC dosages in the soil (Table 1). Among them, the level of Cu and Mn in the leaves of seedlings grown with BC40N0 rose the most; a 125.78 % and 54.32 % (*P* < 0.05) increase was observed compared to those seedlings subjected to neither BC treatment nor N deposition. The BC amendment had the least effect on the accumulation of Fe: it only increased the Fe content by 20.79 % and 6.15 % (*P* < 0.05) with BC20 and BC40 treatments, respectively (Table 1). Compared to the effect of treating the seedlings solely with BC, the accumulation of metal elements did not improve much by treating the seedlings solely with N; moreover, some elements were negatively affected by increased dosages of N. This observation was exemplified by the amount of Ca, Cu, Fe, Mg, Mn and Zn increasing and then decreasing with an increasing dosage of N, while the K content decreased as the N application increased. Nonetheless, combining the treatment of BC and N application, the K content in the seedlings grown with BC20N50 and BC20N150 increased slightly, by 8.51 % and 1.27 % (*P* < 0.05), respectively, compared to those treated with BC20N0; the Fe content in seedlings grown with BC40N50 and BC40N150 increased by 15.55 % and 11.95 % (*P* < 0.05), respectively, compared to those treated with BC40N0.

**Table 1.** Effect of BC amendment with N application on the content of metal elements in the leaves of pecan seedlings. BC0: 0 t ha^−1^ year^−1^; BC20: 20 t ha^−1^ year^−1^; BC40: 40 t ha^−1^ year^−1^; N0: 0 kg N ha^−1^ year^−1^; N50: 50 kg N ha^−1^ year^−1^; N150: 150 kg N ha^−1^ year^−1^. ± represents standard error of three biological replicates. Analysis of variance was based on Duncan’s multiple range test. Lowercase letters indicate significance level of *P* < 0.05.

Treatment	Ca (mg g^−1^)	K (mg g^−1^)	Mg (mg g^−1^)	Cu (mg kg^−1^)	Fe (mg kg^−1^)	Mn (mg kg^−1^)	Zn (mg kg^−1^)
BC0N0	15.10 ± 0.54^d^	14.66 ± 2.60^c^	3.30 ± 0.55^d^	2.25 ± 0.63^de^	87.27 ± 24.88^a^	324.72 ± 19.35^b^	79.23 ± 4.61^cd^
BC0N50	16.86 ± 0.95^bcd^	14.24 ± 0.40^cd^	3.46 ± 0.36^cd^	3.58 ± 0.43^bc^	93.09 ± 9.63^a^	354.16 ± 26.11^b^	78.46 ± 12.25^cd^
BC0N150	15.75 ± 1.12^cd^	11.38 ± 1.58^d^	3.07 ± 0.35^d^	2.03 ± 0.32^e^	87.53 ± 11.69^a^	343.17 ± 32.45^b^	69.28 ± 6.49^d^
BC20N0	19.30 ± 0.69^ab^	16.57 ± 1.07^bc^	4.41 ± 0.35^ab^	3.65 ± 0.47^bc^	105.41 ± 10.97^a^	395.03 ± 36.93^b^	93.65 ± 10.64^abc^
BC20N50	18.52 ± 0.94^ab^	17.98 ± 1.30^ab^	3.78 ± 0.57^bcd^	3.64 ± 0.74^bc^	98.06 ± 16.08^a^	376.53 ± 59.17^b^	83.47 ± 13.52^bcd^
BC20N150	16.98 ± 1.82^bcd^	16.78 ± 1.57^abc^	3.79 ± 0.43^bcd^	3.21 ± 1.14^cd^	99.34 ± 17.12^a^	380.16 ± 31.76^b^	82.58 ± 8.39^bcd^
BC40N0	20.44 ± 2.93^a^	19.92 ± 1.05^a^	4.87 ± 0.39^a^	5.08 ± 0.71^a^	92.64 ± 10.55^a^	501.11 ± 43.71^a^	106.69 ± 13.67^a^
BC40N50	18.76 ± 1.57^ab^	18.69 ± 2.91^ab^	4.53 ± 0.70^ab^	4.30 ± 0.30^abc^	107.05 ± 8.99^a^	487.26 ± 42.77^a^	100.00 ± 6.85^ab^
BC40N150	18.16 ± 0.85^abc^	18.19 ± 1.26^ab^	4.23 ± 0.29^abc^	4.70 ± 0.42^ab^	103.71 ± 15.27^a^	479.46 ± 46.69^a^	92.60 ± 6.47^abc^

### Interaction between BC and N application on the physicochemical properties of soil and seedling growth

Two-way ANOVA analysis (Table 2) showed that BC amendment significantly affected most of the tested parameters except for the stem diameter and Fe content in the leaves (*P* < 0.05); whereas N application has a significant impact on the soil inorganic nitrogen (NH_4_^+^-N, NO_3_^−^-N) content and S-ACP activity, plant height, photosynthesis, chlorophyll content and N element in the leaves (*P* < 0.05). The combined treatment of BC amendment and N application significantly affected the *A*, Chl, Chla and Chlb in the leaves.

**Table 2.** Analysis of interaction between BC and N application on the physicochemical properties of soil, growth index of pecan seedlings and nutrient elements in leaves. Two-way analysis of variance was based on Duncan’s multiple range test. ^†^Indicates significance level of *P* < 0.05.

Parameters	BC		N application		BC * N application	
	*F*	*P*	*F*	*P*	*F*	*P*
Soil bulk density	38.83	0.0000003^†^	0.78	0.4713857	0.11	0.9769807
Soil pH	24.79	0.0000067^†^	2.11	0.1497338	0.23	0.9153230
NH_4_^+^-N	13.77	0.0002350^†^	14.49	0.0001780^†^	0.25	0.9029214
NO_3_^−^-N	11.79	0.0005344^†^	15.46	0.0001237^†^	0.63	0.6482108
S-ACP activity	25.43	0.0000057^†^	5.07	0.0179206^†^	0.45	0.7730760
S-CL activity	22.45	0.0000129^†^	2.15	0.1459948	0.42	0.7951010
S-SC activity	22.45	0.0000129^†^	2.15	0.1459950	0.42	0.7951011
S-UE activity	30.82	0.0000015^†^	1.22	0.3185392	0.82	0.5303909
Plant height	7.47	0.0043471^†^	9.19	0.0017806^†^	1.18	0.3531684
Stem diameter	0.17	0.8439382	3.43	0.0548542	1.12	0.3772480
*A*	25.01	0.0000064^†^	18.85	0.0000385^†^	3.80	0.0207017^†^
*g*_s_	8.82	0.0021400^†^	11.73	0.0005487^†^	2.14	0.1181138
*C*_i_	9.07	0.0018852^†^	4.59	0.0245116^†^	0.58	0.6787288
*E*	6.41	0.0079106^†^	5.60	0.0128738^†^	0.90	0.4857315
Car	5.18	0.0166934^†^	4.04	0.0354604^†^	0.92	0.4745619
Chl	38.73	0.0000003^†^	39.95	0.0000002^†^	9.91	0.0002028^†^
Chla	39.31	0.0000003^†^	45.42	0.0000001^†^	11.27	0.0000900^†^
Chlb	31.28	0.0000014	22.02	0.0000146^†^	6.12	0.0027304^†^
N	6.40	0.0079451^†^	21.83	0.0000154^†^	1.00	0.4351221
P	39.42	0.0000003^†^	2.91	0.0805245	1.21	0.3409694
Ca	11.92	0.0005044^†^	2.12	0.1485922	1.50	0.2442032
K	24.64	0.0000070^†^	2.56	0.1049030	1.11	0.3832540
Mg	17.08	0.0000695^†^	2.65	0.0977304	0.60	0.6647518
Cu	24.83	0.0000067^†^	1.63	0.2240727	2.69	0.0644633
Fe	1.12	0.3491317	0.79	0.4694700	0.70	0.6048298
Mn	34.04	0.0000008^†^	0.06	0.9411067	0.39	0.8098963
Zn	13.80	0.0002331^†^	3.24	0.0627840	0.24	0.9100163

## Discussion

Nutrient elements, such as N, P, K and other microelements, are essential for the growth of plant biomass and yield. Increasing the availability of N via N deposition will facilitate acceleration of plant growth (Galloway *et al.* 2008). Moreover, it has been shown that the application of BC as a soil modifier increases N retention and nutrient cycling by both free-living and symbiotic diazotrophs, which affected nutrient and enzyme dynamic in the soil (Rondon *et al.* 2007; Jones *et al.* 2011). Combining the BC amendment and N application will impose additive effects that will lead to the promotion of plant growth and yield. The impact of such a practice, however, depends on soil condition and plant inherent responses; thus, a detailed study is essentially needed. The present work investigated the effect of BC amendment under varying levels of N application on the growth of pecans. We found that the pecan seedlings benefited the most from the application of a low level of BC, BC20, combined with a high level of N, N150.

In the absence of BC treatment, soil acidification was caused by increasing N deposition, whereas a pH close to neutral could be obtained via increasing BC content in the soil. BC amendment also reduced soil bulk density, indicating it was effective at aerating packed soil. The lower the soil bulk density, the lower the mechanical resistance to root growth; thus, higher root tissue mass density and fine root yield could be obtained, which eventually leads to enhanced nutrient absorption (Backer *et al.* 2017). Moreover, soil permeability and water retention capacity could be improved via lowering soil bulk density. Taken together, through BC amendment, soil fertility and enzyme activity of cellulase, invertase and urease were improved, indicating the advancement of carbon and N cycling, nutrient availability and utilization efficiency in the soil.

When treating the seedlings solely with N, a low dosage of N could improve plant fitness, while a high dosage of N halted seedling growth. In the absence of BC amendment, we found that a low level of N application was effective at increasing the seedling height, whereas the growth of plant height was halted by a high level of N application, suggesting soil acidification, compact soil and the consequence changes in microbial composition that lead to reduction in soil fertility (Gundersen and Rasmussen 1990; Jia *et al.* 2012). This notion was supported by the reduction of cellulase, invertase and urease by BC0N150, which negatively impacted carbon and N cycling in soil. The application of BC alleviated negative effect of a high dosage N application, indicating the mitigation effect of BC amendment on N deposition. Moreover, the combined treatment of BC amendment and N application further improved the seedling height and its photosynthetic ability to the level higher than those grown solely with either BC or N treatment, indicating the additive effect of employing both treatments to promote pecan growth.

Photosynthesis is the basis of plant organic matter synthesis, energy storage and transformation, and chlorophyll content is an important parameter reflecting the photosynthetic function of leaves (Zhang *et al.* 2011). N supplementation directly affects the photosynthetic pigment content in leaves as well as the activity of ribulose 1,5-bisphosphate carboxylase, the enzyme in leaves essential for atmospheric CO_2_ fixation (Makino *et al.* 2000). Furthermore, soil acidification may lead to the adsorption of alkaline cations, disruption of nutrient balance and inhibition of photosynthesis (Bergkvist and Folkeson 1992; Zhang *et al.* 2016). The present work found that with a low level of N application in the absence of BC amendment, the photosynthetic pigments Car, Chla and Chlb increased, whereas a high level of N application suppressed the accumulation of the pigments. The addition of BC improved the N availability and utilization by the seedlings, and this was evidenced by the enhancement of Chl and Car pigments, as well as the photosynthetic rate in the leaves of seedlings.

Despite the elevation of N content and enzymatic activity in the soil correlating positively with the application of BC, a significant level of nitrogen accumulation was only detected in the seedlings treated with BC40. Despite that, we did not detect promotion of growth and photosynthetic ability in the BC40-treated seedlings compared to those treated with BC20. Instead, we found that the seedlings grew better with BC20 amendment and N150 deposition. The BC20-treated seedlings exhibited similar level of accumulated nitrogen as those grown with non-BC treatment, suggesting that the nitrogen absorbed through foliar spraying or soil application was being used quickly for plant development. Further experiments will be needed to investigate how this nutrient uptake and usage is maintained in plants and the mechanisms involve nitrogen allocation from root to leaf and from leaf to leaf, at both the genetic and metabolic level.

Besides the effective use of N in the seedlings treated with BC and N deposition, we found that a high level of N deposition suppressed the accumulation of P in the leaves. Nonetheless, the application of BC alleviated the negative effect of N deposition, wherein the accumulated level of P in the leaves treated with a high level of nitrogen (N150) was raised to a similar level of that under a low level of N50 treatment. The enzymatic activity of acid phosphatase was negatively affected by BC addition to the soil, indicating involvement of other types of phosphatase, such as alkaline phosphatase, in the mechanism of P cycling, which responds to BC amendment, and the BC treatment may have stimulated P transport in the seedlings. After being applied to the soil, the BC supplements a large amount of nutrients, forming a stable nutrient pool for plant growth and development. However, the N application significantly reduced the K content in the leaves of pecan seedlings. The application of micronutrient rich BC was effective at restoring the level of K as well as other micronutrients, such as Ca, Mg, Zn, Cu and Mn in the seedlings.

## Conclusion

Under long-term application of BC amendment and N application, the plant height, chlorophyll content, photosynthesis rate and accumulation of N, P and microelements such as Fe and K were improved in pecan seedlings. The effect of BC amendment and N deposition was additive, and treatment with a lower level of BC (BC20) and a high level of N deposition (N150) was optimum for seedling growth. The agronomic management of pecan plantations requires a careful planning of fertilizer application, which is performed according to the developmental phases of pecan plants. The present work could serve as an important basis for fertilizer management, which could incorporate the application of BC with N application, thereby reducing the use of fertilizer in the field.

## Supporting Information

The following additional information is available in the online version of this article—

Table S1. Plant height and stem diameter of pecan seedlings treated with biochar amendment and nitrogen deposition.

Table S2. Effect of biochar amendment with nitrogen deposition on the (A) intercellular CO_2_ concentration (*C*_i_), (B) stomatal conductance (*g*_s_), (C) net photosynthetic rate (*A*) and (D) transpiration rate (*E*) of the pecan seedlings.

Table S3. The (A) carotenoid (Car), (B) total chlorophyll (Chl), (C) chlorophyll a (Chla) and (D) chlorophyll b (Chlb) content in the pecan leaves over 1 year of treatment combining biochar amendment and nitrogen application.

Table S4. Effect of biochar amendment with nitrogen deposition on the content of (A) carotenoid, (B) total chlorophyll, (C) chlorophyll a and (D) chlorophyll b in the leaves of pecan seedlings.

Table S5. Effect of biochar amendment with nitrogen deposition on the content of (A) nitrogen (N) and (B) phosphorus (P) in the leaves of pecan seedlings.

plaa035_suppl_Supplementary_Tables
