# Supplementary material for: The additive effect of biochar amendment and simulated nitrogen deposition stimulates the plant height, photosynthesis and accumulation of NPK in pecan (Carya illinoinensis) seedlings
Source: AoB Plants. 2020 Jul 26;12(4):plaa035. doi: 10.1093/aobpla/plaa035 (PMC7441530; doi:10.1093/aobpla/plaa035)
Supplement: plaa035_suppl_Supplementary_Tables [file plaa035_suppl_supplementary_tables.pdf]

Supporting Information - Table S1 Plant height and stem diameter of pecan seedlings treated with biochar amendment and nitrogen deposition. BC0: 0t ha^{-1} y^{-1}; BC20: 20t ha^{-1} y^{-1}; BC40: 40t ha^{-1} y^{-1}; N0: 0kg N ha^{-1} y^{-1}; N50: 50kg N ha^{-1} y^{-1}; N150: 150kg N ha^{-1} y^{-1}. ± represents standard errors of three biological replicates. Analysis of variance was based on Duncan's multiple range test. Lowercase letters indicate significance level of $p < 0.05$.

Treatment	Plant height （cm）	Stem diameter (cm)
BC0N0	115.89 ± 3.69^{e}	1.14 ± 0.13^{a}
BC0N50	133.44 ± 2.25^{bcde}	1.26 ± 0.47^{a}
BC0N150	127 ± 7.09^{cde}	1.34 ± 0.42^{a}
BC20N0	121.33 ± 4.67^{de}	1.31 ± 0.6^{a}
BC20N50	137.06 ± 5.72^{abcd}	1.18 ± 0.89^{a}
BC20N150	148.22 ± 7.73^{ab}	1.33 ± 0.88^{a}
BC40N0	134.67 ± 5.24^{bcd}	1.18 ± 1.02^{a}
BC40N50	137 ± 7.47^{abc}	1.28 ± 0.49^{a}
BC40N150	152.89 ± 2.16^{a}	1.36 ± 0.43^{a}

Supporting Information - Table S2 Effect of biochar amendment with nitrogen deposition on the (A) intercellular CO_2 concentration (C_i), (B) stomatal conductance (g_s), (C) net photosynthetic rate (A), and (D) transpiration rate (E) of the pecan seedlings. BC0: 0t ha^{-1} y^{-1}; BC20: 20t ha^{-1} y^{-1}; BC40: 40t ha^{-1} y^{-1}; N0: 0kg N ha^{-1} y^{-1}; N50: 50kg N ha^{-1} y^{-1}; N150: 150kg N ha^{-1} y^{-1}. ± represents standard errors of three biological replicates. Analysis of variance was based on Duncan's multiple range test. Lowercase letters indicate significance level of $p < 0.05$.

Treatment	C_i(μmol m^{-2} s^{-1})	g_s(mol m^{-2} s^{-1})	A(μmol m^{-2} s^{-1})	E(mmol m^{-2} s^{-1})
BC0N0	183.61 ± 26.78^{a}	0.042 ± 0.005^{d}	4.17 ± 0.23^{f}	1.63 ± 0.21^{c}
BC0N50	164.06 ± 7.42^{abc}	0.059 ± 0.004^{cd}	5.06 ± 0.20^{de}	1.94 ± 0.14^{abc}
BC0N150	170.92 ± 20.15^{ab}	0.045 ± 0.006^{d}	4.43 ± 0.39^{ef}	1.76 ± 0.18^{bc}
BC20N0	176.30 ± 17.80^{a}	0.047 ± 0.015^{d}	4.50 ± 0.81^{ef}	1.75 ± 0.49^{bc}
BC20N50	157.26 ± 24.36^{abcd}	0.069 ± 0.016^{bc}	5.45 ± 0.46^{cd}	2.08 ± 0.25^{abc}
BC20N150	133.09 ± 10.21^{bcd}	0.080 ± 0.008^{ab}	6.32 ± 0.39^{ab}	2.34 ± 0.16^{a}
BC40N0	148.60 ± 27.28^{abcd}	0.055 ± 0.011^{cd}	5.14 ± 0.44^{de}	1.97 ± 0.24^{abc}
BC40N50	128.77 ± 21.51^{cd}	0.072 ± 0.010^{abc}	5.92 ± 0.65^{bc}	2.21 ± 0.27^{ab}
BC40N150	120.19 ± 16.10^{d}	0.087 ± 0.006^{a}	6.83 ± 0.31^{a}	2.42 ± 0.19^{a}

Supporting Information - Table S3 The (A) carotenoid (Car), (B) total chlorophyll (Chl), (C) chlorophyll a (Chla), and (D) chlorophyll b (Chlb) content in the pecan leaves over one year of treatment combining biochar amendment and nitrogen application. BC0: 0t ha^{-1} y^{-1}; BC20: 20t ha^{-1} y^{-1}; BC40: 40t ha^{-1} y^{-1}; N0: 0kg N ha^{-1} y^{-1}; N50: 50kg N ha^{-1} y^{-1}; N150: 150kg N ha^{-1} y^{-1}. ± represents standard errors of three biological replicates. Analysis of variance was based on Duncan's multiple range test. Lowercase letters indicate significance level of $p < 0.05$.

A) Carotenoid (Car)

	2017.5	2017.7	2017.9	2107.11	2018.5
BC0N0	0.67 ± 0.05^{ab}	0.89 ± 0.10^{a}	0.61 ± 0.06^{c}	0.60 ± 0.06^{c}	0.53 ± 0.11^{c}
BC0N50	0.67 ± 0.09^{ab}	0.87 ± 0.18^{a}	0.68 ± 0.11^{bc}	0.72 ± 0.14^{bc}	0.58 ± 0.09^{abc}
BC0N150	0.71 ± 0.07^{ab}	0.90 ± 0.07^{a}	0.80 ± 0.20^{abc}	0.80 ± 0.09^{abc}	0.54 ± 0.11^{bc}
BC20N0	0.65 ± 0.02^{b}	0.98 ± 0.14^{a}	0.87 ± 0.11^{ab}	0.66 ± 0.13^{c}	0.52 ± 0.09^{c}
BC20N50	0.72 ± 0.08^{ab}	0.96 ± 0.07^{a}	0.91 ± 0.14^{a}	0.73 ± 0.17^{bc}	0.60 ± 0.08^{abc}
BC20N150	0.78 ± 0.03^{a}	1.00 ± 0.09^{a}	0.96 ± 0.14^{a}	0.90 ± 0.08^{ab}	0.72 ± 0.08^{ab}
BC40N0	0.72 ± 0.07^{ab}	0.91 ± 0.13^{a}	0.98 ± 0.08^{a}	0.76 ± 0.11^{bc}	0.60 ± 0.09^{abc}
BC40N50	0.6 ± 0.07^{b}	0.97 ± 0.10^{a}	0.91 ± 0.10^{a}	0.88 ± 0.14^{ab}	0.73 ± 0.11^{a}
BC40N150	0.74 ± 0.06^{ab}	0.94 ± 0.08^{a}	1.01 ± 0.09^{a}	1.00 ± 0.15^{a}	0.75 ± 0.09^{a}

B) Total chlorophyll (Chl)

	2017.5	2017.7	2017.9	2107.11	2018.5
BC0N0	2.38 ± 0.66^{a}	3.73 ± 0.44^{b}	2.60 ± 0.16^{e}	1.74 ± 0.27^{d}	2.09 ± 0.07^{d}
BC0N50	2.55 ± 0.94^{a}	3.66 ± 0.31^{b}	2.87 ± 0.40^{de}	2.13 ± 0.28^{cd}	2.39 ± 0.15^{cd}
BC0N150	2.88 ± 0.45^{a}	3.80 ± 0.09^{b}	3.31 ± 0.15^{cd}	2.48 ± 0.54^{bc}	2.17 ± 0.10^{cd}
BC20N0	2.15 ± 0.04^{a}	3.85 ± 0.29^{b}	3.54 ± 0.20^{bc}	2.11 ± 0.36^{cd}	2.06 ± 0.09^{d}
BC20N50	2.55 ± 0.20^{a}	4.01 ± 0.27^{ab}	3.86 ± 0.48^{abc}	2.47 ± 0.21^{bc}	2.45 ± 0.14^{c}
BC20N150	3.00 ± 0.25^{a}	4.17 ± 0.26^{ab}	3.99 ± 0.44^{ab}	2.99 ± 0.39^{ab}	3.31 ± 0.46^{a}
BC40N0	3.04 ± 0.59^{a}	4.06 ± 0.52^{ab}	3.94 ± 0.44^{ab}	2.52 ± 0.20^{bc}	2.48 ± 0.10^{c}
BC40N50	2.40 ± 0.18^{a}	4.55 ± 0.30^{a}	4.09 ± 0.20^{ab}	2.93 ± 0.35^{ab}	2.99 ± 0.09^{b}
BC40N150	2.82 ± 0.19^{a}	4.49 ± 0.32^{a}	4.37 ± 0.24^{a}	3.27 ± 0.18^{a}	3.52 ± 0.15^{a}

C) Chlorophyll a (Chla)

	2017.5	2017.7	2017.9	2107.11	2018.5
BC0N0	1.62 ± 0.56^{a}	2.64 ± 0.22^{b}	1.92 ± 0.12^{e}	1.20 ± 0.17^{d}	1.43 ± 0.06^{d}
BC0N50	1.74 ± 0.74^{a}	2.58 ± 0.23^{b}	2.10 ± 0.27^{de}	1.50 ± 0.15^{cd}	1.71 ± 0.11^{c}
BC0N150	2.03 ± 0.33^{a}	2.69 ± 0.04^{b}	2.41 ± 0.09^{cd}	1.81 ± 0.38^{bc}	1.51 ± 0.06^{cd}
BC20N0	1.42 ± 0.06^{a}	2.61 ± 0.13^{b}	2.57 ± 0.14^{bc}	1.54 ± 0.20^{cd}	1.41 ± 0.04^{d}
BC20N50	1.73 ± 0.10^{a}	2.79 ± 0.03^{ab}	2.73 ± 0.30^{abc}	1.81 ± 0.13^{bc}	1.71 ± 0.12^{c}
BC20N150	2.02 ± 0.16^{a}	2.91 ± 0.16^{ab}	2.81 ± 0.28^{abc}	2.20 ± 0.27^{ab}	2.39 ± 0.34^{a}
BC40N0	2.14 ± 0.47^{a}	2.83 ± 0.29^{ab}	2.81 ± 0.26^{abc}	1.86 ± 0.15^{bc}	1.75 ± 0.09^{c}
BC40N50	1.64 ± 0.15^{a}	3.12 ± 0.15^{a}	2.92 ± 0.18^{ab}	2.10 ± 0.23^{ab}	2.10 ± 0.07^{b}
BC40N150	1.94±0.15a	3.13 ± 0.21^{a}	3.08 ± 0.17^{a}	2.34±0.13a	2.55 ± 0.09^{a}

D) Chlorophyll b (Chlb)

	2017.5	2017.7	2017.9	2107.11	2018.5
BC0N0	0.74 ± 0.11^{b}	1.05 ± 0.22^{b}	0.67 ± 0.05^{d}	0.53 ± 0.10^{d}	0.64 ± 0.02^{b}
BC0N50	0.79 ± 0.19^{ab}	1.05 ± 0.08^{b}	0.75 ± 0.15^{cd}	0.62 ± 0.14^{bcd}	0.66 ± 0.04^{b}
BC0N150	0.83 ± 0.12^{ab}	1.08 ± 0.05^{ab}	0.87 ± 0.08^{cd}	0.66 ± 0.16^{bcd}	0.64 ± 0.04^{b}
BC20N0	0.71 ± 0.02^{b}	1.20 ± 0.17^{ab}	0.94 ± 0.07^{bc}	0.56 ± 0.16^{cd}	0.64 ± 0.06^{b}
BC20N50	0.80 ± 0.10^{ab}	1.20 ± 0.25^{ab}	1.10 ± 0.18^{ab}	0.64 ± 0.08^{bcd}	0.71 ± 0.02^{b}
BC20N150	0.96 ± 0.09^{a}	1.23 ± 0.10^{ab}	1.15 ± 0.16^{ab}	0.76 ± 0.12^{ab}	0.89 ± 0.11^{a}
BC40N0	0.88 ± 0.12^{ab}	1.19 ± 0.23^{ab}	1.10 ± 0.18^{ab}	0.65 ± 0.05^{bcd}	0.71 ± 0.01^{b}
BC40N50	0.74 ± 0.04^{b}	1.40 ± 0.15^{a}	1.14 ± 0.04^{ab}	0.80 ± 0.12^{ab}	0.86 ± 0.02^{a}
BC40N150	0.86 ± 0.04^{ab}	1.33 ± 0.11^{ab}	1.26 ± 0.07^{a}	0.91 ± 0.06^{a}	0.95 ± 0.06^{a}

Supporting Information - Table S4 Effect of biochar amendment with nitrogen deposition on the content of (A) carotenoid, (B) total chlorophyll, (C) chlorophyll a and (D) chlorophyll b in the leaves of pecan seedlings. BC0: 0t ha^{-1} y^{-1}; BC20: 20t ha^{-1} y^{-1}; BC40: 40t ha^{-1} y^{-1}; N0: 0kg N ha^{-1} y^{-1}; N50: 50kg N ha^{-1} y^{-1}; N150: 150kg N ha^{-1} y^{-1}. ± represents standard error of three biological replicates. Analysis of variance was based on Duncan's multiple range test. Lowercase letters indicate significance level of $p < 0.05$.

Treatment	Car content （mg g^{-1}）	Chla content （mg g^{-1}）	Chlb content （mg g^{-1}）	Chl content （mg g^{-1}）
BC0N0	0.53 ± 0.11^{c}	1.43 ± 0.06^{d}	0.64 ± 0.02^{b}	2.09 ± 0.07^{d}
BC0N50	0.58 ± 0.09^{abc}	1.71 ± 0.11^{c}	0.66 ± 0.04^{b}	2.39 ± 0.15^{cd}
BC0N150	0.54 ± 0.11^{bc}	1.51 ± 0.06^{cd}	0.64 ± 0.04^{b}	2.17 ± 0.10^{cd}
BC20N0	0.52 ± 0.09^{c}	1.41 ± 0.04^{d}	0.64 ± 0.06^{b}	2.06 ± 0.09^{d}
BC20N50	0.60 ± 0.08^{abc}	1.71 ± 0.12^{c}	0.71 ± 0.02^{b}	2.45 ± 0.14^{c}
BC20N150	0.72 ± 0.08^{ab}	2.39 ± 0.34^{a}	0.89 ± 0.11^{a}	3.31 ± 0.46^{a}
BC40N0	0.60 ± 0.09^{abc}	1.75 ± 0.09^{c}	0.71 ± 0.01^{b}	2.48 ± 0.10^{c}
BC40N50	0.73 ± 0.11^{a}	2.10 ± 0.07^{b}	0.86 ± 0.02^{a}	2.99 ± 0.09^{b}
BC40N150	0.75 ± 0.09^{a}	2.55 ± 0.09^{a}	0.95 ± 0.06^{a}	3.52 ± 0.15^{a}

Supporting Information - Table S5 Effect of biochar amendment with nitrogen deposition on the content of (A) nitrogen (N) and (B) phosphorus (P) in the leaves of pecan seedlings. BC0: 0t ha^{-1} y^{-1}; BC20: 20t ha^{-1} y^{-1}; BC40: 40t ha^{-1} y^{-1}; N0: 0kg N ha^{-1} y^{-1}; N50: 50kg N ha^{-1} y^{-1}; N150: 150kg N ha^{-1} y^{-1}. ± represents standard errors of three biological replicates. Analysis of variance was based on Duncan's multiple comparison. Lowercase letters indicate significance level of $p < 0.05$.

treatment	N content **(mg g^{-1})**	P content **(mg g^{-1})**
BC0N0	18.78 ± 1.06^{c}	2.35 ± 0.06^{c}
BC0N50	19.23 ± 0.36^{c}	2.18 ± 0.06^{cd}
BC0N150	24.05 ± 0.88^{ab}	2.06 ± 0.06^{d}
BC20N0	19.05 ± 1.04^{c}	2.47 ± 0.1^{bc}
BC20N50	19.32 ± 0.59^{c}	2.28 ± 0.09^{cd}
BC20N150	24.93 ± 0.62^{ab}	2.36 ± 0.05^{c}
BC40N0	23.34 ± 1.16^{ab}	2.85 ± 0.15^{a}
BC40N50	$21.30 \pm .48^{bc}$	2.70 ± 0.06^{ab}
BC40N150	25.61 ± 1.33^{a}	2.90 ± 0.11^{a}
